# Exploring multielement nanogranular coatings to forestall implant-related infections

**DOI:** 10.3389/fcimb.2023.1128822

**Published:** 2023-02-07

**Authors:** Marta Bottagisio, Vincenzo Balzano, Luca Ciambriello, Laura Rosa, Giuseppe Talò, Arianna B. Lovati, Elena De Vecchi, Luca Gavioli

**Affiliations:** ^1^ IRCCS Istituto Ortopedico Galeazzi, Laboratory of Clinical Chemistry and Microbiology, Milan, Italy; ^2^ Interdisciplinary Laboratories for Advanced Materials Physics (i-LAMP), Dipartimento di Matematica e Fisica, Università Cattolica del Sacro Cuore, Via Musei, Brescia, Italy; ^3^ IRCCS Istituto Ortopedico Galeazzi, Cell and Tissue Engineering Laboratory, Milan, Italy

**Keywords:** implant-related infections, biofilm, biomaterials, orthopedics, Mg-Ag-Cu and Ti-Ag-Cu nanoparticles, bioactive coatings, nanogranular coatings

## Abstract

**Introduction:**

As we approach the post-antibiotic era, the development of innovative antimicrobial strategies that carry out their activities through non-specific mechanisms could limit the onset and spread of drug resistance. In this context, the use of nanogranular coatings of multielement nanoparticles (NPs) conjugated to the surface of implantable biomaterials might represent a strategy to reduce the systemic drawbacks by locally confining the NPs effects against either prokaryotic or eukaryotic cells.

**Methods:**

In the present study, two new multielement nanogranular coatings combining Ag and Cu with either Ti or Mg were synthesized by a gas phase physical method and tested against pathogens isolated from periprosthetic joint infections to address their potential antimicrobial value and toxicity in an *in vitro* experimental setting.

**Results:**

Overall, *Staphylococcus aureus, Staphylococcus epidermidis* and *Escherichia coli* displayed a significantly decreased adhesion when cultured on Ti-Ag-Cu and Mg-Ag-Cu coatings compared to uncoated controls, regardless of their antibiotic resistance traits. A dissimilar behavior was observed when Pseudomonas aeruginosa was cultured for 30 and 120 minutes upon the surface of Ti-Ag-Cu and Mg-Ag-Cu-coated discs. Biofilm formation was mainly reduced by the active effect of Mg-Ag-Cu compared to Ti-Ag-Cu and, again, coatings had a milder effect on *P. aeruginosa*, probably due to its exceptional capability of attachment and matrix production. These data were further confirmed by the evaluation of bacterial colonization on nanoparticle-coated discs through confocal microscopy. Finally, to exclude any cytotoxic effects on eukaryotic cells, the biocompatibility of NPs-coated discs was studied. Results demonstrated a viability of 95.8% and 89.4% of cells cultured in the presence of Ti-Ag-Cu and Mg-Ag-Cu discs, respectively, when compared to negative controls.

**Conclusion:**

In conclusion, the present study demonstrated the promising anti-adhesive features of both Ti-Ag-Cu and Mg-Ag-Cu coatings, as well as their action in hampering the biofilm formation, highlighting the safe use of the tested multi-element families of nanoparticles as new strategies against bacterial attachment to the surface of biomedical implants.

## 1 Introduction

The era of biomaterials able to restore the loss of mechanical tissue function has deeply conditioned modern medicine and orthopedic clinical practice, contributing to the improvement of the quality of life in an increasingly elderly population (Ratner, 2019). Despite prophylaxis, the main complication associated with the massive use of orthopedic devices is infection, jeopardizing their integration and function and frequently requiring an invasive procedure to replace the contaminated implants to overcome the pathological status (Zimmerli et al., 2004).

The insertion of biocompatible materials into the host offers microbes the ideal substrate on which to adhere and arrange colonies in a sessile community protected by an extracellular polymeric substance (EPS), known as biofilm (Del Pozo, 2018). The biofilm state is an evolutionary advantage that confers bacteria defense from the host immune system attacks and antibiotic therapy, masking their presence upon implants. Furthermore, within the EPS matrix, sessile bacteria enter a quiescence state, slowing down their metabolism while perfectly coordinating their vital functions with the other microorganisms and causing indolent and chronic biofilm infections (Le et al., 2018). An inactive metabolism not only guarantees a longer bacterial survival, but it is also an extremely powerful weapon against many classes of antibiotics, making the treatment ineffective (Stokes et al., 2019).

To discourage the rise of this dangerous scenario, novel bioactive materials have been extensively studied. The key goal is the development of a biomaterial able to support the integration of the implanted device while hampering bacterial adhesion and biofilm growth upon its surface, possibly without the use of antibiotics. The main limit in the use of antibiotic-loaded biomaterials is the potential development/acquisition of antibiotic resistance traits. Indeed, if contaminants can survive the initial massive antibiotic release, the chances of acquiring resistance traits increase exponentially over time because of the scarcer antibiotic leakage (Stanton et al., 2020). Hence, to avoid any risky drawbacks, many efforts have been devoted to the development of coatings able to interfere with the early stages of biofilm formation by preventing bacterial attachment and/or killing the bacteria.

Coatings based on metal and metal oxide nanoparticles (NPs) are renowned for their antimicrobial effects (Baranwal et al., 2018; Benetti et al., 2019; Bottagisio et al., 2019; Benetti et al., 2020). The different materials and the multiple biotic targets result in a material-specific killing activity comprising in some cases selected Gram-positive and Gram-negative bacteria, including drug-resistant strains (Wang et al., 2017). Furthermore, the synthesis method of the deposited layer becomes important when the goal is to widen the antimicrobial spectrum, but at the same time to avoid high levels of metal content to limit undesired toxic side effects. In this perspective, the NPs loading amount and the relative elemental concentration inside the nanogranular building blocks are of paramount importance (Hu et al., 2014; Benetti et al., 2017; Benetti et al., 2019) and are usually provided in µg/cm^2^ for coatings obtained by physical deposition methods. Typical values for magnetron sputtered coatings are ranging from 75 µg/cm^2^ for Ag supported on boron nitride (Gudz et al., 2022), to 210 µg/cm^2^ for a mixed Ag/TiAlVN compound (Marín et al., 2022) and up to 2000 µg/cm^2^ for AgCo/Fe(Al, Cr)2O_4_ microstructure (Xu et al., 2022). When atomic layer deposition is employed, the metal amount changes from 157 µg/cm^2^ for Ag/TiO_2_ nanostructures (Nazarov et al., 2022) down to a few µg/cm^2^ for AgNP deposited on a steel surface (González et al., 2021). For nanostructured coatings obtained by gas phase beams pure silver (Ag) (Cavaliere et al., 2015), AgTiO_2_ (Benetti et al., 2017), and Mg-Ag-Cu (Benetti et al., 2019) composite the loading varies from 10 to 2 µg/cm^2^. These numbers suggest that the latter method is much more suited for targeting low coating toxicity, even by combining more than one active element inside the film. In particular, Benetti and colleagues in 2019 showed the possibility of combining at least three different elements in a single NP, and proved the developed Mg-Ag-Cu coating to be effective against *Escherichia coli* ATCC 25922 and *Staphylococcus aureus* ATCC 6538, respectively (Benetti et al., 2019). However, this promising coating was not investigated with respect to bacterial adhesion and biofilm formation.

Since it is well known that the physical properties of a nanoparticles-based coating such as the NPs size distribution and stoichiometry, the coating morphology, and the amount of loaded metals are influencing the antimicrobial response, a number of questions are still open. The antimicrobial effect or the presence of other oxygen-affine materials in the NPs composites, the actual efficacy against bacteria adhesion and biofouling remains yet to be investigated. Finally, targeting undefined microbiological mechanisms could be a double-edged sword, interfering with the physiological role of eukaryotic cells too. Hence, it is important to establish and respect the concentration window that regulates the use of NPs according to their effects, searching for an equilibrium between microorganisms kill and the detrimental effects on host cells (Flores et al., 2013). These aspects make the quest for wide-spectrum antimicrobial coatings with an effective mixing of different active materials a still open challenge, particularly for orthopedic implants. Starting from the promising results of the Mg-Ag-Cu case, in the present study, we pursued the goal of effective mixing of different active materials by investigating the antimicrobial properties of two multielement nanogranular coatings composed of Ti-Ag-Cu and Mg-Ag-Cu. The use of elements with high oxygen affinity such as Ti and Mg should allow for maintaining the Ag and Cu metallicity, which is important for the antibacterial efficacy of these elements. The coatings were synthesized by employing the SCBD, an eco-friendly synthesis technique with high throughput, and tested against pathogens isolated from periprosthetic joint infections to address their potential antimicrobial value in an *in vitro* experimental setting.

## 2 Materials and methods

### 2.1 Deposition of Ti-Ag-Cu and Mg-Ag-Cu films

Ti-Ag-Cu and Mg-Ag-Cu NPs were deposited on circular cover glasses (VWR) by Supersonic Cluster Beam Deposition (SCBD), a physical method that exploits pulsed high-pressure Helium (He) injections in a low vacuum (10-3 mbar) chamber and synchronized electric discharges to ignite a plasma that homogeneously erodes all the constituents of the target rod. The quenched atoms then condense into NPs and are extracted through an aerodynamic system, forming a beam towards the substrate. As in previous works (Benetti et al., 2019; Benetti et al., 2020; Valerini et al., 2020; Balzano et al., 2021), a motorized rastering system allowed the deposition of a uniform film on multiple samples at the same time, allowing the uniformity of the coating thickness on all the samples provided for the microbiological tests. The nominal thickness of the films was monitored *via* a quartz microbalance and measured separately by atomic force microscopy (AFM). The nominal atomic composition of the rods (Hauner Gmbh) was 78/10/12 at% for the Mg-Ag-Cu rod and 71/11/18 at% for the Ti-Ag-Cu rod. The weight ratio was chosen to obtain a similar atomic fraction of Ag and a higher atomic fraction of Cu in the coatings.

The NP size and shape distributions produced by SCBD were obtained from AFM data, analyzing sparse NPs deposited both on a Silicon (Si) substrate (0.1 nm RMS roughness). The set of AFM (Park NX10) images was acquired in air and at room temperature in tapping mode with a PPP-NCSTR tip (Nanosensor, resonance frequency 120-250 Hz, nominal tip radius 10nm), with 0.25 pixels/nm. The distributions of the NP heights were elaborated by Gwyddion software (Nečas and Klapetek, 2012).

The film composition was evaluated by X-Ray photoelectron spectroscopy (XPS) in a multiscan system (Omicron) using an Mg X-Ray source (incident photon energy was 1253.6 eV) and a Phoibos 100 SPECS analyzer at 20 eV pass energy. Data were acquired on 40 nm thick films deposited onto a Si substrate. The corresponding photoelectron sampling depth for the elements composing the film is up to 5 nm. The data were analyzed using CasaXPS software, (Copyright © 2005 Casa Software Ltd) by employing GL(30) peaks for all the non-metal components.

### 2.2 Bacterial strains

Clinically relevant strains isolated from implant-related infections at the Laboratory of Clinical Chemistry and Microbiology of the IRCCS Galeazzi Orthopedic Institute were used in the present investigation. In particular, biofilm-producer strains of *Staphylococcus aureus* (MSSA and MRSA), *Staphylococcus epidermidis* (MSSE and MRSE), *Escherichia coli* (S and R), and *Pseudomonas aeruginosa* (S and R) with different antibiotic susceptibility profile were selected.

Microbiological identification was performed at a phenotypic level by Vitek2 System (BioMeriéux). Antimicrobial susceptibility testing and determination of the minimum inhibitory concentration (MIC) were carried out by Vitek2 System using the card AST-P659 for staphylococci and AST-N376 for Gram-negative bacteria (all BioMeriéux).

### 2.3 Evaluation of bacterial adhesion on nanoparticles-coated discs

The anti-adhesive properties of coatings characterized by different NPs compositions (Ti-Ag-Cu and Mg-Ag-Cu) were tested. Uncoated circular cover glasses (VWR) were used as growth control (CTRL).

Briefly, a bacterial suspension of 1.0 × 10^7^ CFU/ml was prepared in Brain Heart Infusion broth (BHI, BioMeriéux) supplemented with 50% v/v human sterile serum (Merck). Thereafter, three discs per experimental condition (CTRL or Ti-Ag-Cu or Mg-Ag-Cu) were placed in a Petri dish and 100 µl of the bacterial suspension were dispensed on the surfaces of the coated or uncoated disks exploiting the surface tension of the liquid. Two independent tests were performed in triplicate for each strain. All the experimental procedures were carried out in the dark. After 30 and 120 minutes of incubation at 37°C, disks were rinsed twice with sterile saline to remove any non-adherent cells. Then, disks were placed in a sterile 5 ml tube containing 1 ml of 0.1% dithiothreitol solution (DTT, Sigma-Aldrich) (Drago et al., 2013) and sonicated at 45 kHz for 5 minutes to both mechanically and chemically dislodge bacteria. The eluates were then serially diluted and drop-plated (Herigstad et al., 2001) on tryptic soy agar (TSA, Sigma-Aldrich) plate and incubated at 37°C for 24 hours. Thereafter, viable colonies were counted and the number of colony forming units (CFU) was recorded.

### 2.4 Evaluation of bacterial biofilm formation on nanoparticles-coated discs

The biofilm-forming ability of the tested clinical isolates on discs coated either with Ti-Ag-Cu or Mg-Ag-Cu was evaluated by means of a spectrophotometric assay as previously reported (Christensen et al., 1985; Bidossi et al., 2019).

Discs were cultured in 900 μl of BHI broth (BioMeriéux) supplemented with 50% v/v human sterile serum (Merck) and 100 μl of 1.0 × 10^7^ CFU/ml bacterial suspension were incubated for 24 hours at 37°C under a mild agitation (250 rpm). At the end of the incubation, discs were rinsed three times with sterile saline solution to remove any non-adherent cells and let them dry to fix the biofilm biomass. Once dried, each disc was stained with 100 μl of a 1% crystal violet solution (Merck) for 10 minutes and washed again with sterile saline solution to remove any dye excess. As previously described, the 100-μl-dye was placed only on the treated/upper surface of the discs exploiting the surface tension of the liquid to avoid any aspecific stain. After the complete drying of the matrix, discs were placed in new 12-well plates and 1 ml absolute ethanol was added to each well to solubilize the crystal violet staining the biofilm matrix. The amount of biomass was then quantified spectrophotometrically by reading at 595 nm wavelength 100 μl of the extracted dye solution by means of a microplate reader (Multiskan FC, ThermoFisher Scientific). Two independent experiments were run in triplicates for each experimental condition. All the experimental procedures were carried out in the dark.

### 2.5 Evaluation of bacterial colonization on nanoparticles-coated discs

The bacterial colonization of the surface of discs either coated or uncoated with nanoparticles was evaluated by means of confocal laser scanning microscopy (CLSM) as previously reported (Bidossi et al., 2018).

Discs were placed in a 12-well microplate with 900 μl of BHI broth (BioMeriéux) and 100 μl of 1.0 × 10^7^ CFU/ml bacterial suspension. After 24 hours of incubation at 37°C under a mild agitation (250 rpm), discs were rinsed three times with sterile saline to remove non-adherent bacteria and 100 μl of dying solution composed of 3 μl of SYTO9 in 1 ml of filter-sterilized water (Thermo Fisher Diagnostics) were dispensed on the surfaces of discs. Samples were incubated at room temperature in the dark for 15 minutes. Thereafter, discs were washed again with sterile saline to remove any dye excess and examined with an upright TCS SP8 (Leica Microsystems CMS GmbH) using a 20× dry objective (HC PL FLUOTAR 20×/0.50 DRY). Images from at least three randomly selected areas were acquired for each sample; two independent investigations were performed for each experimental condition.

The obtained images were processed with Las X (Leica Microsystems CMS GmbH) and finally analyzed with Fiji software (Fiji, ImageJ, Wayne Rasband National Institutes of Health). Briefly, a custom macro has been developed in Fiji, and a “Z project” was performed for the channel. The channel was then thresholded and the total area of live cells was evaluated using “measure”.

### 2.6 Evaluation of the biocompatibility of nanoparticles-coated discs

The biocompatibility of nanoparticle-coated discs was studied to exclude any cytotoxic effect on eukaryotic cells. Briefly, 3 x 10^5^ NIH-3T3 murine fibroblasts (ATCC^®^ CRL-1658)/cm^2^ were seeded in 6-well plates and cultured in complete medium (CM) consisting of Dulbecco’s Modified Eagle’s Medium (DMEM, ATCC^®^ 30-2002), 10% calf bovine serum (ATCC^®^ 30-2030™), 100 U/ml penicillin-streptomycin, 2 mM L-glutamine, 1% sodium pyruvate, 1% HEPES (all from Gibco, Life Technologies). After a 24-hours at 37°C and 5% CO_2_, three Ti-Ag-Cu or Mg-Ag-Cu discs were placed in a Transwell (Corning) for an indirect co-culture with the underlying cell monolayer. Cells cultured in fresh CM served as the negative control (NC) and fibroblast cultured in CM supplemented with 0.1% Triton-X100 (Sigma-Aldrich) were used as positive control (PC). After 24 hours, cell viability was assessed by the MTT assay. Briefly, cell monolayers were washed three times with phosphate-buffered saline (PBS, Gibco, Life Technologies), then 1 m of MTT in DMEM without phenol red (0.5 mg MTT/ml) was added to each well and incubated at 37°C and 5% CO_2_ for 2 hours. Thereafter, the MTT solution was gently removed and the formazan crystals solubilized with 500 μl of a 1:10 solution of hydrochloric acid 1N in isopropanol (both Sigma-Aldrich). The absorbance was read at 570 nm by means of a microplate reader (Victor X3, Perkin Elmer). Two independent experiments were run in triplicates for each experimental condition. All the experimental procedures were carried out in the dark.

### 2.7 Statistical analysis

Data were recorded from each microbiological evaluation derived from two independent experiments both performed in triplicate for each bacterial strain and experimental conditions. The normal distribution of data was evaluated with the Shapiro-Wilk test. Comparisons among groups were performed by the Kruskal-Wallis test followed by Dunn’s multiple comparison test (Graph Pad Prism v8.00 Software Inc., San Diego, CA, USA). A two-way ANOVA coupled with Bonferroni’s *post hoc* test was performed to analyze the effect of nanoparticles-coated discs on bacterial adhesion over time. All data are expressed as means ± standard deviation (SD). Values of p < 0.05 were considered statistically significant.

## 3 Results

### 3.1 Nanostructured coating characterization

**Figure 1:**
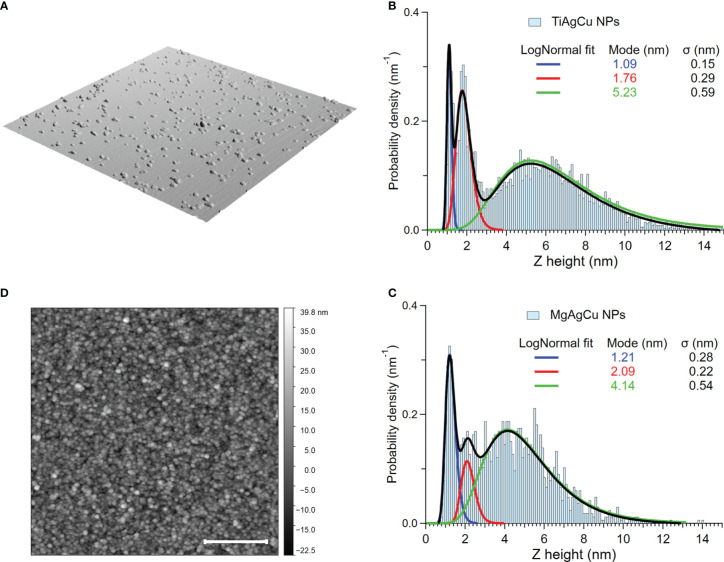
Morphological characterization of the NPs and of the films resulting from the NP deposition. **(A)** Three-dimensional representation of a typical 2x2 µm^2^ AFM image taken on sparse NPs of both compounds deposited on a smooth Si(100) surface. These data were used to obtain the histograms reported in panels **(B)** and **(C)**. **(B)** Distribution of the Ti-Ag-Cu NP height, with the three-modal fit obtained with lognormal distributions and the corresponding modes. **(C)** Distribution of the Mg-Ag-Cu NP height, with the three-modal fit obtained with lognormal distributions and the corresponding modes. **(D)** Typical 4x4 µm^2^ AFM image taken on the 12 nm thick coating. Scale bar 1µm.

The coatings were obtained by depositing the NPs on the substrate from the supersonic beam. The NPs aggregation behavior was expected to follow the ballistic deposition regime (Milani and Iannotta, 1999; Binns, 2001; Borghi et al., 2018), hence the knowledge of the individual NP size distribution is of fundamental importance. **Figure 1** describes the morphology of both the Ti-Ag-Cu and Mg-Ag-Cu coatings. The NPs size was obtained by analyzing AFM images similar to those shown in **Figure 1A**, where it is possible to identify each NP and extract the corresponding vertical height, which is not influenced by tip convolution effects and hence provides the true NP height. Comparison of the histograms of **Figures 1B, C**, evidence for both compounds a three-modal distribution, with slightly different values of the modes for each compound, in particular for the larger NPs (5.2 ± 0.59 nm for Ti-Ag-Cu and 4.1 ± 0.54 nm for Mg-Ag-Cu, respectively).

The surface morphology of the coatings is well described by the AFM image reported in **Figure 1D**, collected on the 12 nm thick films employed for all the microbiological tests. The NPs forming the coating are uniformly distributed, giving rise to the nanogranular morphology. This behavior is a common feature for films obtained by SCBD, independently from the substrate and from the material composing the NPs (Cavaliere et al., 2017; Borghi et al., 2018; Benetti et al., 2019; Benetti et al., 2020), due to the soft landing regime resulting from the synthesis conditions (Milani and Iannotta, 1999; Binns, 2001). The root mean square (RMS) roughness obtained from several topographic images taken in different film areas is (3.7 ± 0.4) nm for Ti-Ag-Cu and 5.2 ± 0.4 nm for Mg-Ag-Cu.

**Figure 2:**
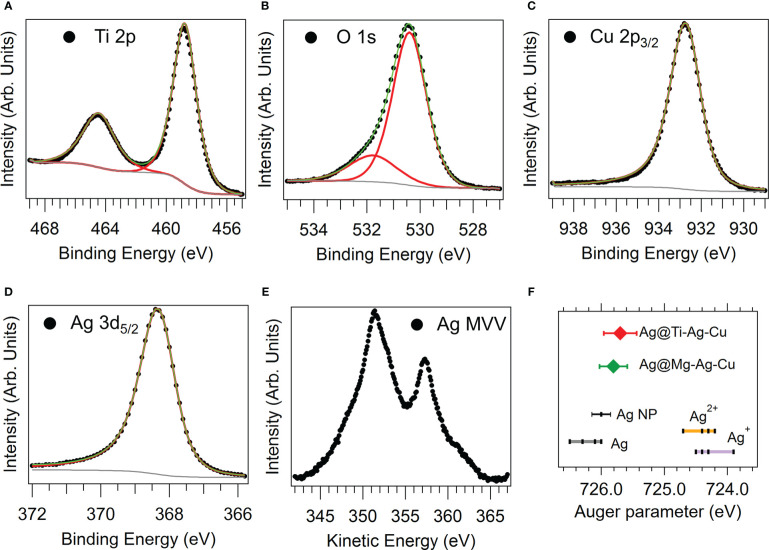
Spectroscopic characterization of the Ti-Ag-Cu sample. **(A)** Ti 2p, **(B)** O1s, **(C)** Cu 2p (only the 2p_3/2_ component is shown), **(D)** Ag 3d (only the 3d_5/2_ component is shown), and **(E)** Ag MVV Auger emission spectra. **(F)** Scheme of the Auger parameters obtained for the Ti-Ag-Cu (red diamond) and Mg-Ag-Cu (green diamond) samples.

Since the chemical state of Ag, Cu, Mg, and Ti influences their bactericidal activity (Ruparelia et al., 2008; Webster and Seil, 2012; Wang et al., 2017), a thorough XPS analysis has been carried out on both coatings. In **Figure 2** the results obtained for the Ti-Ag-Cu are reported. The XPS core level peaks were fitted employing mixed Lorentzian-Gaussian functions with a certain degree of asymmetry for the metallic components. The binding energy (BE) positions, full width at half maximum (FWHM), and atomic concentrations obtained from the fitting procedure are reported in **Table 1**, for future reference. All the elements present in the rod employed in the ablation step are present in the nanostructured film. The Ti 2p (458.78 eV, **Figure 2A**) and the O1s (530.4 eV **Figure 2B**) BE, together with their relative 1:2 atomic concentration reported in **Table 1**, indicate the presence of TiO_2_, with Ti in the 4+ configuration state. An identical behavior was observed for bi-elemental Ag-Ti NP obtained by SCBD (Benetti et al., 2017). Concerning Cu, the absence of satellites typical of oxidized Cu (Kumari and Majewski, 2013) and the Cu 2p3/2 peak BE (932.7 eV, **Figure 2C**) clearly indicate the metallic state (Wagner, 1979) of the element within the NP. In the case of Ag, one has to consider the Ag 3d peak BE (368.3), the MVV Auger lineshape (Benetti et al., 2017), and the Auger parameter (see **Figures 2D–F**, respectively). The latter allows for comparison with literature results for metallic (Ag) and oxidex (Ag^+^, Ag^2+^) silver (Waterhouse et al., 2002; Ferraria et al., 2012) and for pure Ag NPs obtained by SCBD (Benetti et al., 2017), indicating the metallicity of Ag in the Ti-Ag-Cu film. The XPS data obtained on the Mg-Ag-Cu 12 nm-thick film are in good agreement with previously reported data obtained on an 85 nm thick film deposited by SCBD in the same experimental conditions.

**Table 1:** The binding energy (BE) positions, full width at half maximum (FWHM), and atomic concentrations of Ti-Ag-Cu obtained from the fitting procedure.

Peak	BE ± 0.8 (eV)	W ± 0.2 (eV)	At ± 1 %	At* ± 1 %	At** ± 1 %	Peak	BE ± 0.8 (eV)	W ± 0.2 (eV)	At ± 1 %	At* ± 1 %	At** ± 1 %
Cu 2p_3/2_	932.7	1.7	8	19	18	Cu 2p_3/2_	932.7	1.5	7	12	10
Ag 3d_5/2_	368.3	1.2	5	12	11	Ag 3d_5/2_	368.3	1.2	10	17	12
O 1_s_	530.4	1.5	58	–	–	O 1_s_	530.1	1.8	42	–	–
Ti 2_p_	458.8	1.8	29	69	71	Mg 2_p_	49.6	1.5	41	71	78

W is the full width at half maximum of each component. At* is the relative atomic concentration excluding oxygen. At** is the nominal relative atomic concentration as deduced from the supplier mass weight of the rods.

The relative atomic concentrations obtained from the experiment are reported in **Table 1**. With respect to the nominal ratios, very similar concentrations for most of the elements were observed, except for a slightly higher Ag content in the Mg-Ag-Cu case. These results confirm the suitability of the SCBD method to prepare nanostructured films of compounds with tailorable stoichiometry.

### 3.2 Bacterial strain characterization

Biofilm-producer strains of *S. aureus* (MSSA and MRSA), *S. epidermidis* (MSSE and MRSE), *E. coli* (S) and (R), and *P. aeruginosa* (S) and (R) were identified and characterized by means of a Vitek2 System. In particular, the antibiotic susceptibility profiles are summarized in **Tables 2** and **3**, respectively.

**Table 2:** Antimicrobial resistance profile of Staphylococcal strains.

Strain	Antibiotics															
	BP	OXA	GM	LVX	ERY	CM	LNZ	DPC	TP	VA	TET	TGC	FOS	FA	RA	SXT
MSSA	>0.25	0.5	≤0.5	≤0.12	1	0.25	2	0.25	≤0.5	1	≤1	≤0.12	≤8	≤0.5	≤0.03	≤10
MRSA	>0.25	>2	>8	>4	>4	>2	2	1	4	2	2	0.5	>64	≤0.5	≤0.03	≤10
MSSE	–	≤0.25	≤0.5	≤0.12	1	0.25	2	0.5	8	1	≤1	≤0.12	≤8	≤0.5	≤0.03	≤10
MRSE	–	>2	≤0.5	≤0.12	>4	0.25	2	0.25	1	1	>8	≤0.12	>64	≤0.5	≤0.03	≤10

BP, benzylpenicillin; OXA, oxacillin; GM, gentamicin; ERY, erythromycin; CM, clindamycin; LNZ, linezolid; DPC, daptomycin; TP, teicoplanin; VA, vancomycin; TET, tetracycline; TGC, tigecycline; FA, fusidic acid; RA, rifampin; SXT, trimethoprim/sulfamethoxazole.

Data are reported as μg/ml.

**Table 3:** Antimicrobial resistance profile of Gram-negative strains. Data are reported as μg/ml.

Strain	Antibiotics														
	IMI	GM	TGC	SXT	AMC	PTZ	CTX	CAZ	FEP	ETP	MEM	AK	CIP	FOS	CLi
*E. coli* (S)	–	≤1	≤0.5	≤20	≤2	≤4	≤4	≤0.12	≤0.12	≤0.12	≤0.25	2	≤0.06	≤16	≤0.5
*E. coli* (R)	≤0.25	>8	≤0.5	>160	16	≤4	>32	16	4	≤0.5	≤0.25	4	>2	≤16	≤0.5
*P. aeruginosa* (S)	–	2	>4	>160	–	8	16	2	–	–	1	4	0.25	>128	≤0.5
*P. aeruginosa* (R)	2	≤1	≥8	80	≥32	≤4	8	≤1	2	–	≤0.25	≤2	≥4	–	≤0.5

IP, imipenem; GM, gentamicin; TGC, tigecycline; SXT, trimethoprim/sulfamethoxazole; AMC, amoxicillin/clavulanic acid; PTZ, piperacillin/tazobactam; CTX, cefotaxime; CAZ, ceftazidime; FEP, cefepime; ETP, ertapenem; MEM, meropenem; AK, amikacin; CIP, ciprofloxacin; FOS fosfomycin; CL, colistin.

### 3.3 Evaluation of bacterial adhesion on nanoparticles-coated discs

**Figure 3:**
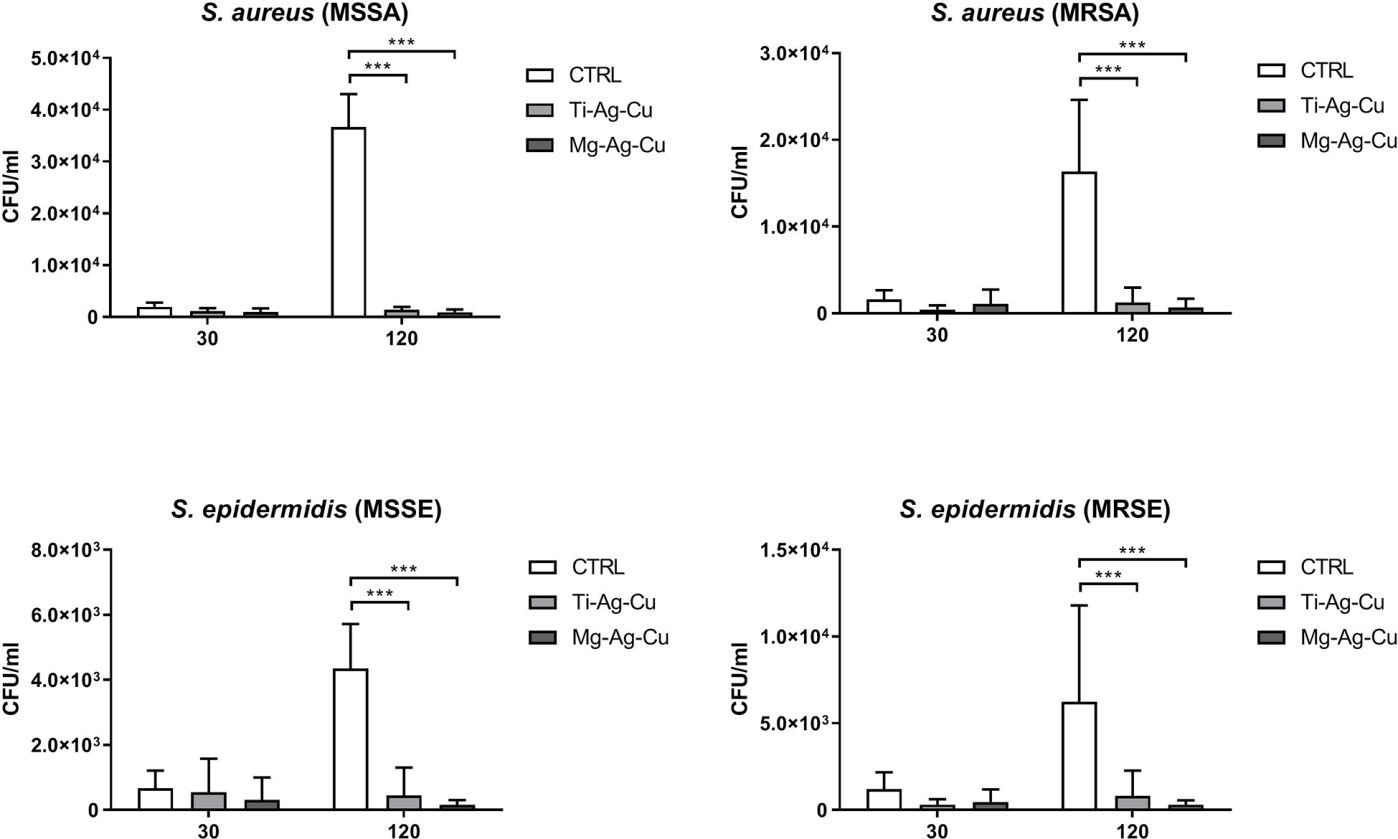
Staphylococcal adhesion on the surface of Ti-Ag-Cu and Mg-Ag-Cu-coated discs and uncoated glass surface (CTRL) after 30 and 120 minutes of incubation. Statistical significance: p < 0.001 (***).

The staphylococcal adhesion on the surface of Ti-Ag-Cu and Mg-Ag-Cu-coated discs after 30 and 120 minutes of incubation is reported in **Figure 3**. It can be noticed that the 30-minute culture was not sufficient to allow the detection of any statistical differences between coated or uncoated discs when analyzed with data collected after a 120 minutes-incubation, due to the greater number of CFU/ml. Differently, at the latest time point, the staphylococcal adhesion on both Ti-Ag-Cu and Mg-Ag-Cu coated discs was significantly lower when compared to uncoated glass surface (p<0.001). A consistent trend was observed throughout all the staphylococcal strains, regardless of the antibiotic resistance traits.

**Figure 4:**
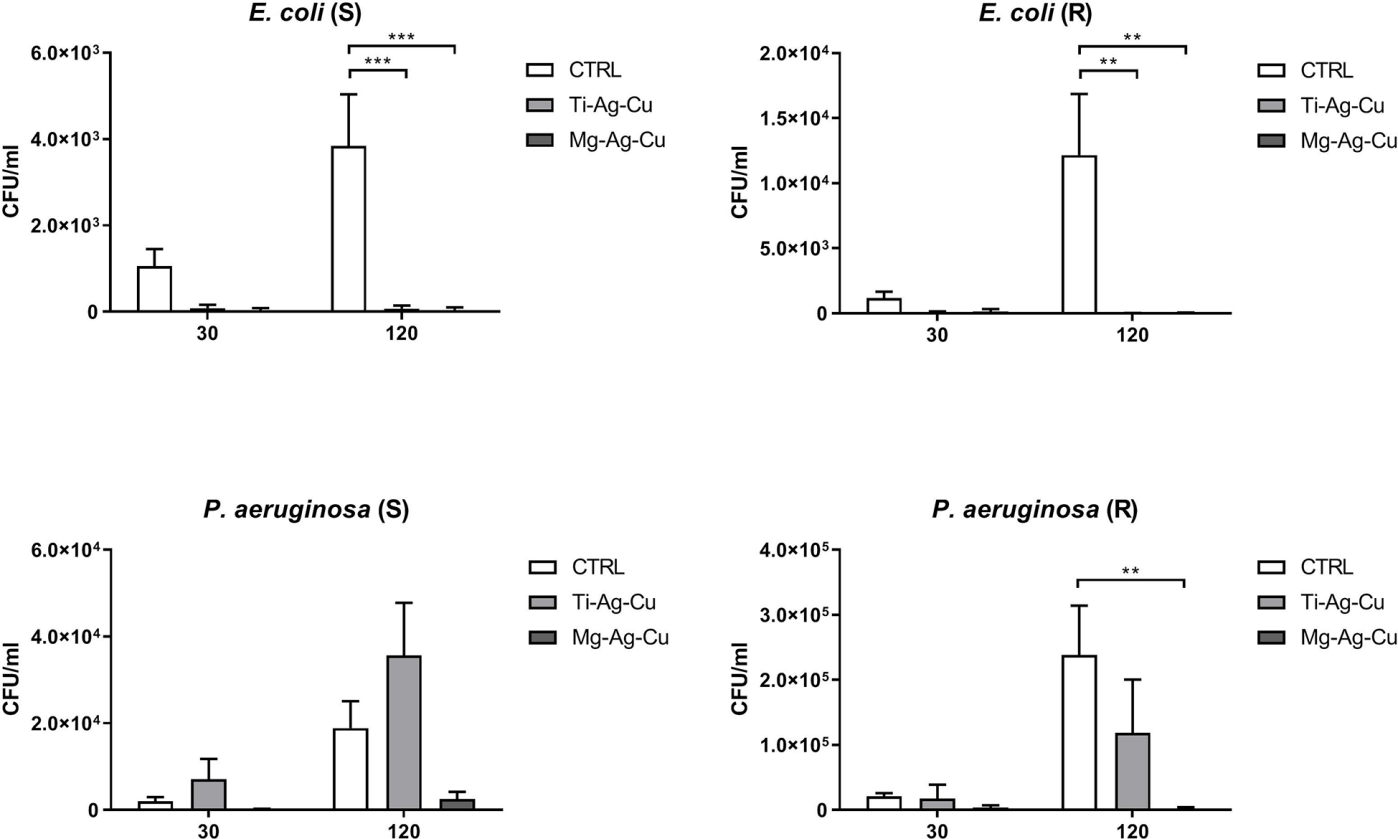
Adhesion of *E. coli* and *P. aeruginosa* on the surface of Ti-Ag-Cu and Mg-Ag-Cu-coated discs and uncoated glass surface (CTRL) after 30 and 120 minutes of incubation. Statistical significance: p < 0.01 (**) and p < 0.001 (***).

**Figure 4** reports the adhesion of *E. coli* and *P. aeruginosa* strains on the surface of Ti-Ag-Cu and Mg-Ag-Cu-coated and CTRL discs after 30 and 120 minutes of incubation. Again, the main differences determined by the treatment of the surface of the discs were detectable after 120 minutes of incubation. For what concerned *E. coli*, both the nanoparticles coating-mixes demonstrated the ability to negatively influence the bacterial attachment compared to the surface of the uncoated disc (p<0.001 for *E.coli* (S) vs Ti-Ag-Cu and Mg-Ag-Cu and p<0.01 for *E.coli* (R) vs Ti-Ag-Cu and Mg-Ag-Cu).

A dissimilar behavior was observed when *P. aeruginosa* was cultured for 30 and 120 minutes upon the surface of Ti-Ag-Cu and Mg-Ag-Cu-coated discs. While the adhesion of *P. aeruginosa* (R) was clearly inhibited by the Mg-Ag-Cu-mixture compared to the uncoated control after a 120-minute incubation (p<0.01), no significant difference was recorded in the number of cells adhering to the surface of Ti-Ag-Cu discs compared to controls. On the other hand, the anti-adhesive ability of both NPs mixtures was not powerful enough to negatively influence the adhesion of *P. aeruginosa* (S), where no significant differences were detected among experimental groups.

### 3.4 Evaluation and quantification of biofilm biomass on nanoparticles-coated discs

**Figure 5:**
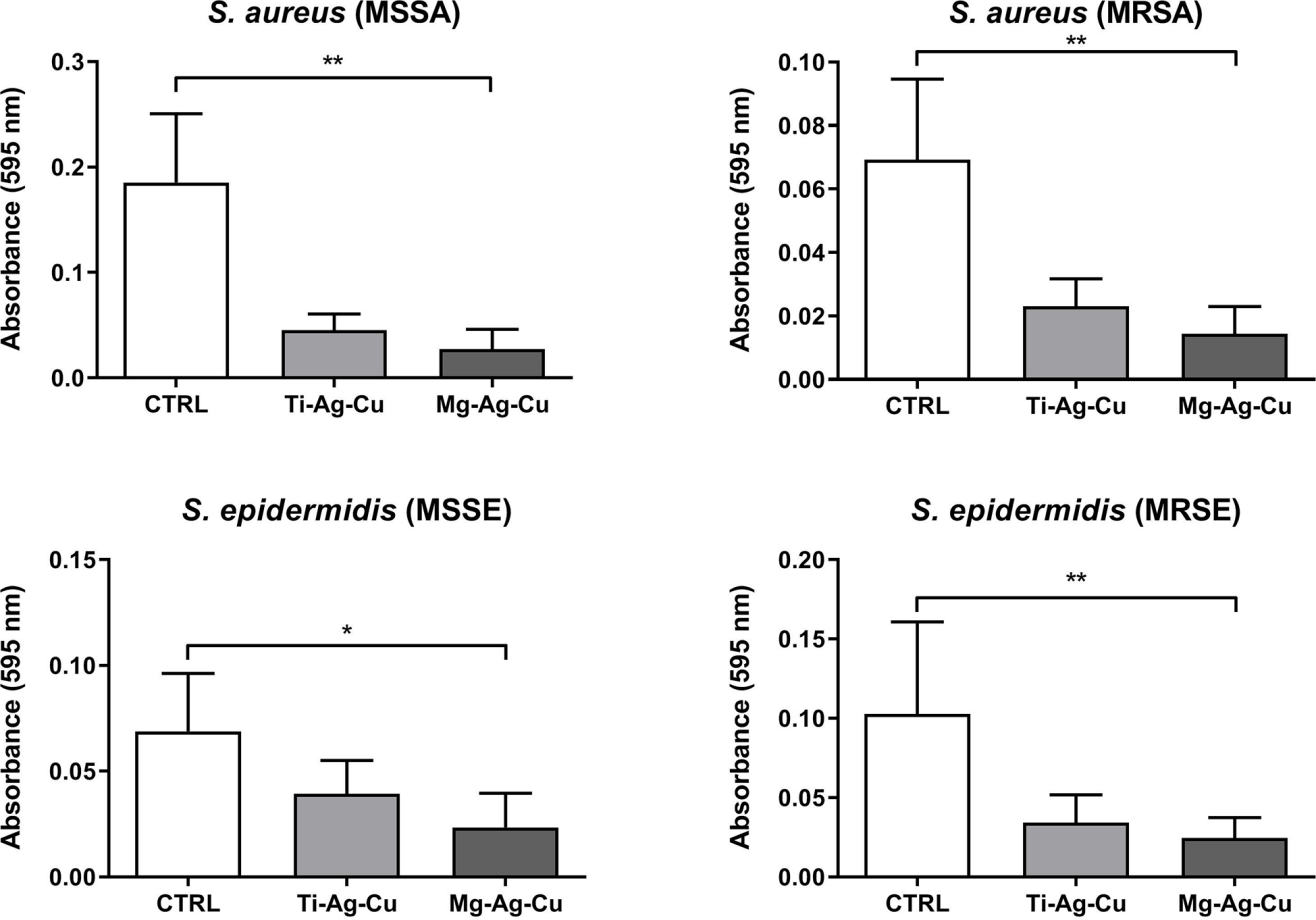
Effect of Ti-Ag-Cu and Mg-Ag-Cu coating on *S. aureus* (MSSA and MRSA) and *S. epidermidis* (MSSE and MRSE) biofilm compared to uncoated glass discs (CTRL) analyzed by means of the spectrophotometric crystal violet assay. Statistical significance: p < 0.05 (*) and p < 0.01 (**).

Through the crystal violet spectrophotometric assay, it was possible to quantify the amount of biofilm biomass deposited by clinical isolates after 24 hours of culture at 37°C. Once again, staphylococci showed a common clear behavior demonstrating a lower affinity to Mg-Ag-Cu-coated surfaces in terms of biofilm deposition (**Figure 5**). Conversely, no differences in the quantity of biofilm biomass were detected when comparing uncoated controls to Ti-Ag-Cu coating.

**Figure 6:**
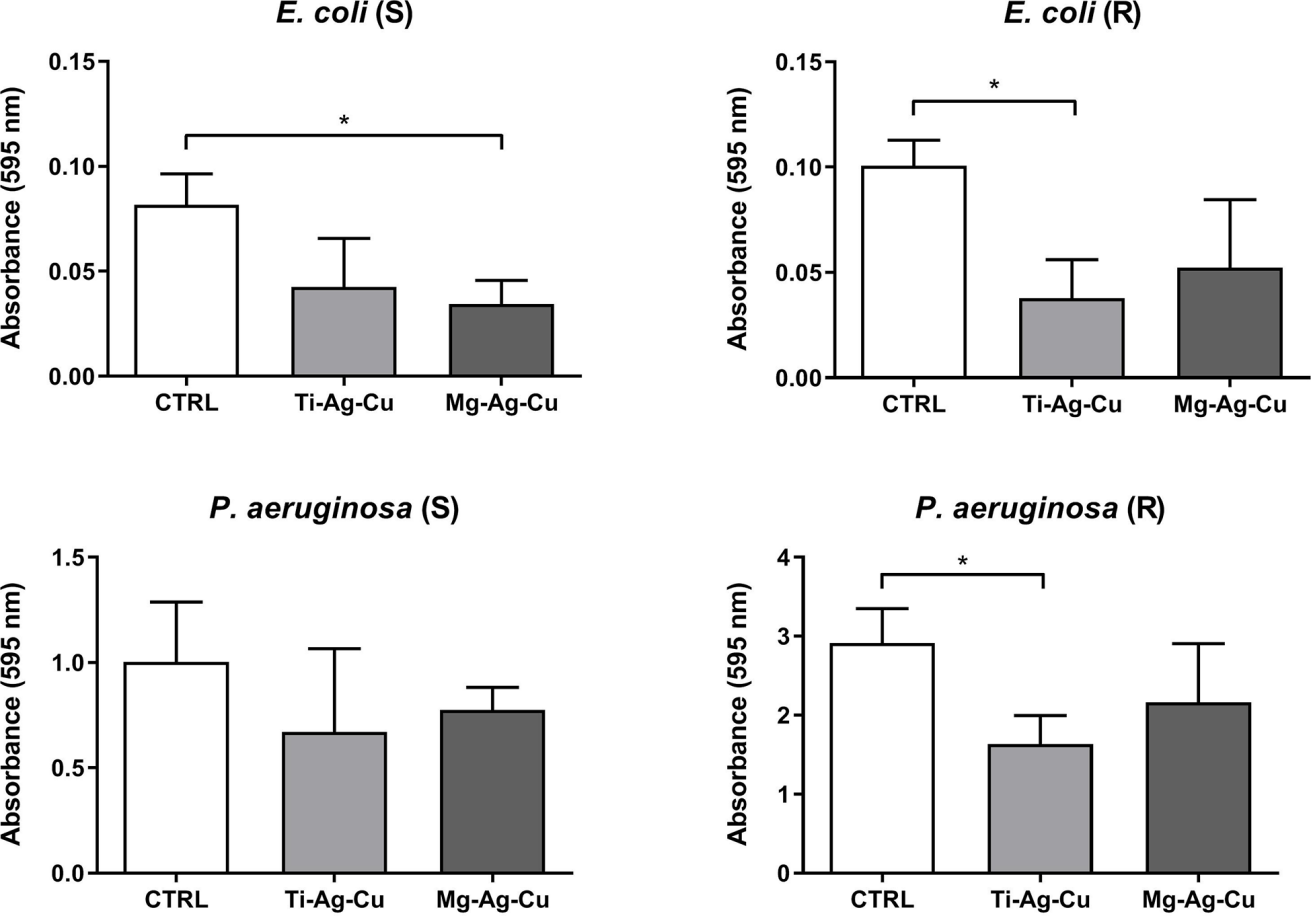
Effect of Ti-Ag-Cu and Mg-Ag-Cu coating on *E. coli* (S and R) and *P. aeruginosa* (S and R) biofilm compared to uncoated glass discs (CTRL) analyzed by means of the spectrophotometric crystal violet assay. Statistical significance: p < 0.05 (*).

Differently, a milder inhibitory effect was observed in the biofilm deposition of Gram-negative clinical isolates on Ti-Ag-Cu and Mg-Ag-Cu coated discs compared to the untreated CTRL (**Figure 6**). In particular, the two *E. coli* isolates demonstrated a different behavior when cultured on Ti-Ag-Cu or Mg-Ag-Cu coated discs. A slightly lower biomass deposition on Mg-Ag-Cu by *E. coli* (S) and on Ti-Ag-Cu by *E. coli* (R) was observed when compared to CTRL. Again, the two NP-coatings did not cause a reduction in the biofilm formation by *P. aeruginosa* (S), whereas the biofilm production by *P. aeruginosa* (R) seemed to be affected by the Ti-Ag-Cu coated discs.

### 3.5 Evaluation of bacterial colonization on nanoparticles-coated discs

**Figure 7:**
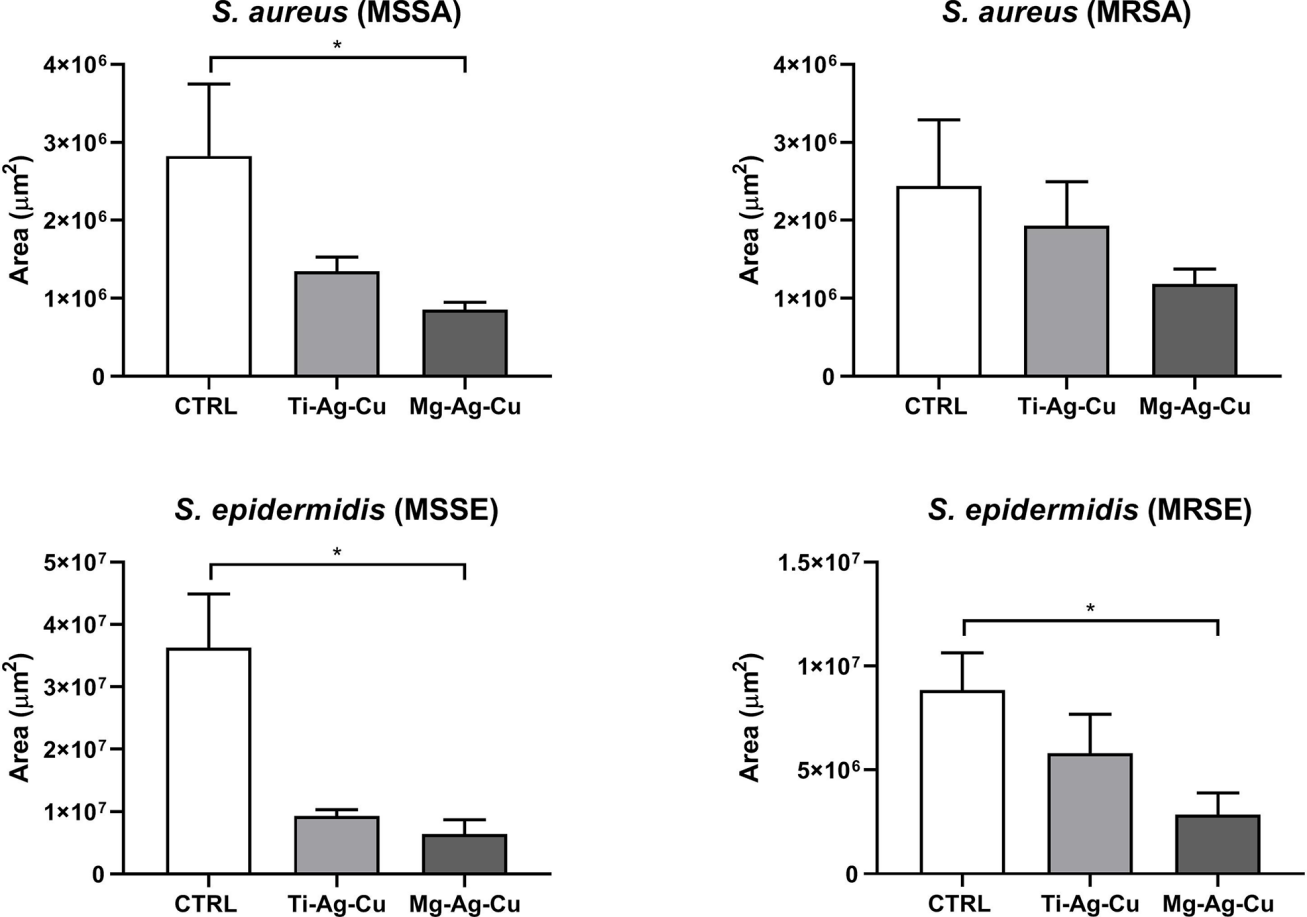
Effect of Ti-Ag-Cu and Mg-Ag-Cu coating S. aureus (MSSA and MRSA) and S. epidermidis (MSSE and MRSE) colonization compared to uncoated glass discs (CTRL) analyzed by means of CLSM. Statistical significance: p < 0.05 (*).

The effects of Ti-Ag-Cu and Mg-Ag-Cu coating on the bacterial colonization of the surface of the disc were evaluated by means of CLSM. The signal detected from dyed living staphylococci complied with the results obtained by the evaluation and quantification of biofilm biomass. Briefly, discs coated with Mg-Ag-Cu discouraged the bacterial attachment to the treated surfaces compared to uncoated CTRL, especially for MSSA, MSSE, and MRSE for a p < 0.05 (**Figure 7**). The effects of the Mg-Ag-Cu coating on MRSA were perceptible, but not statistically significant when compared to MRSA growth on CTRL. Similarly, the activity of Ti-Ag-Cu coating was not sufficient to appreciate any difference from a statistical point of view.

**Figure 8:**
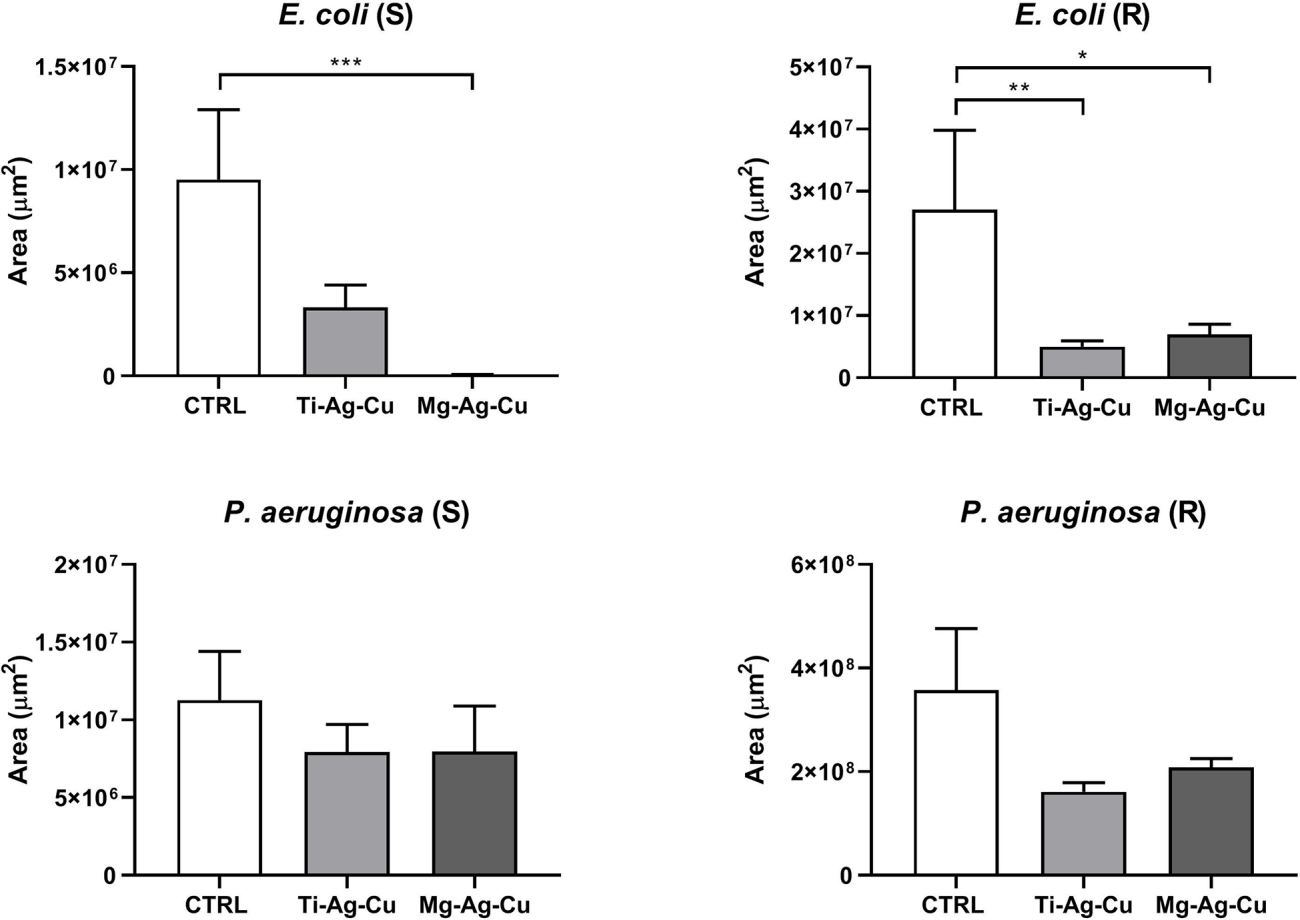
Effect of Ti-Ag-Cu and Mg-Ag-Cu coating on *E. coli* (S and R) and *P. aeruginosa* (S and R) colonization compared to uncoated glass discs (CTRL) analyzed by means of CLSM. Statistical significance: p < 0.05 (*), p < 0.01 (**) and p < 0.001 (***).

For what concerned the growth of Gram-negative bacteria (**Figure 8**), both *E. coli* (S) and (R) demonstrated a lower affinity for Mg-Ag-Cu coated surface compared to uncoated CTRL (p < 0.001 and p <0.05, respectively). Consistently with results obtained by the CV spectrophotometric assay, Ti-Ag-Cu effects against *E. coli* (R) colonization were also highlighted by SYTO9 staining when compared to uncoated CTRL (p < 0.01).

No effects due to both Ti- and Mg-NP coatings were detected when *P. aeruginosa* (S) and (R) were cultured on them.

### 3.6 Evaluation of the biocompatibility of nanoparticles-coated discs

**Figure 9:**
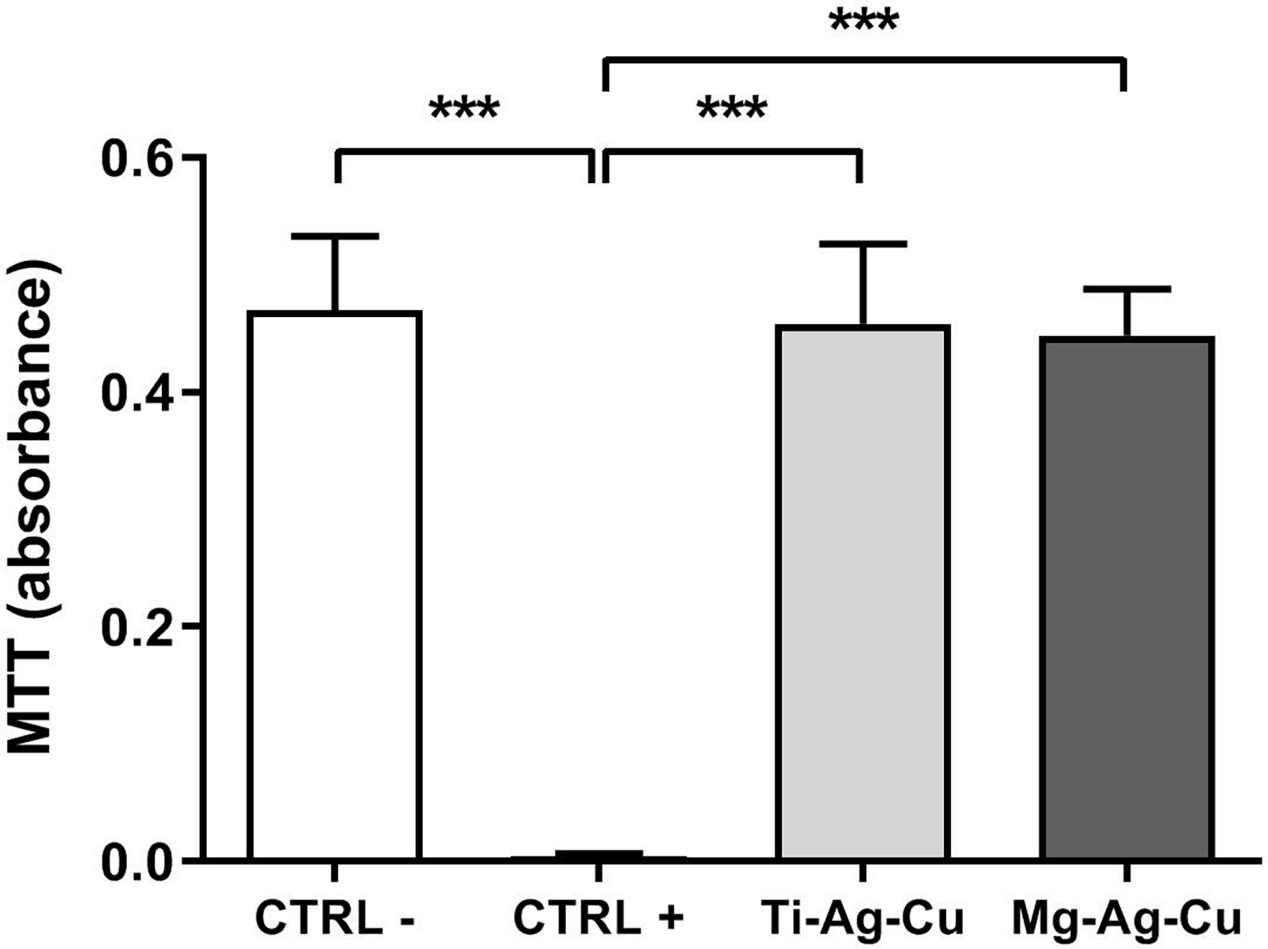
Effect of Ti-Ag-Cu and Mg-Ag-Cu coating on the viability of NIH 3T3 fibroblast compared to cells cultured in CM (CTRL -) and CM supplemented with 0.1% Triton-X100 (CTRL +) assessed by means of the MTT assay. Statistical significance: p < 0.001 (***).

The biocompatibility of nanoparticles-coated discs was assessed by means of the spectrophotometric MTT viability assay. No signs of cytotoxicity were recorded when culturing NIH 3T3-fibrolast in the presence of Ti-Ag-Cu or Mg-Ag-Cu, as illustrated in **Figure 9**. Indeed, absorbance levels of fibroblast indirectly cultured with Ti-Ag-Cu or Mg-Ag-Cu were comparable to those obtained by cells cultured in fresh CM (CTRL-). Consequently, the statistical differences reported in the histogram (**Figure 9**) refer to the comparison between CTRL -, Ti-Ag-Cu, and Mg-Ag-Cu when compared to lysed cells treatment with Triton X-100 (CTRL +). The mitochondrial function assessment by the MTT assay can work as an index to estimate relative cell number in relation to CTRL – and it showed 97.6% and 95.5% of cell viability when cells were indirectly cultured in the presence of Ti-Ag-Cu and Mg-Ag-Cu, respectively.

## 4 Discussion

The widespread use and misuse of antibiotics and the consequent emerge of multidrug-resistant bacterial strains encourage researchers to explore different strategies in the fight against bacterial adhesion and biofilm formation upon implantable medical devices. In this context, the broad-spectrum antibacterial properties of metal-derived NPs against both Gram-positive and Gram-negative bacteria are well documented in the literature, making them promising candidates in the fight against infections, especially in the orthopedic field (Bottagisio et al., 2019). To further broaden the spectrum of action against bacteria, coatings composed of different combinations of metal NPs (Ti-Ag-Cu and Mg-Ag-Cu) have been designed and tested in the present work. Silver and copper NPs were selected because of their solid and documented antibacterial effects, increasing the spectrum of action on as many infectious agents as possible (Wang et al., 2017). Conversely, titanium and magnesium were introduced to mitigate the potential cytotoxic damages caused by Ag and Cu NPs and to improve the adhesion of the coating to the substrate. For this study, *S. aureus*, *S. epidermidis*, *E. coli*, and *P. aeruginosa* isolated from periprosthetic joint infections were used, being the Gram-positive and Gram-negative species most commonly involved in this type of complication (Drago et al., 2017).

It was first evaluated the ability of the aforementioned clinical isolates to adhere to Ti-Ag-Cu and Mg-Ag-Cu coated discs, simulating *in vitro* the first contact of bacteria with the implanted material. Indeed, adhesion is the first step of the biofilm formation process upon implants and it involves several molecular and physical interactions leading to cell accumulation and EPS matrix production. Staphylococcal species, that together account for almost 50% of all PJIs (Aggarwal et al., 2014; Drago et al., 2017), always commonly displayed a significantly decreased attachment to Ti-Ag-Cu and Mg-Ag-Cu coated discs after 120 minutes of contact, with respect to untreated discs. No appreciable differences between strains and antibiotic resistance traits were detected. A similar behavior was recorded for *E. coli* isolates, while Ti-Ag-Cu and Mg-Ag-Cu coatings had a milder effect on the attachment of *P. aeruginosa* strains, which owns an exceptional capability of attachment and matrix production (McConoughey et al., 2014; Bidossi et al., 2020). Furthermore, differences in biofilm quantification were also appreciated by the detection of all the organic matrix compounds by the crystal violet spectrophotometric assay and through the quantification of the cell biomass by CLSM analysis (Azeredo et al., 2017). The longer the culture of the bacterial strains, the milder the effects of the Ti-Ag-Cu and Mg-Ag-Cu coatings, underlying a greater action of the NPs-based coatings in the very first stage of bacterial interactions. Hence, the overall antimicrobial results of the nanostructured coatings suggested that the Mg-Ag-Cu film provided a better response again the tested strains, in particular for the adhesion assay.

This may suggest a role of the higher Ag concentration in the Mg-based film with respect to the Ti-based coating. It is worth noting that, in the framework of minimizing the toxic metal amounts in the coating, the present results are very promising since the films present a Cu and Ag loading lower than 5 µg/cm^2^, falling in the lower extreme range of the amount previously reported for coatings obtained by physical deposition methods (Benetti et al., 2017; Benetti et al., 2020; Valerini et al., 2020; González et al., 2021). Furthermore, the less effective action of the coatings in the biofilm and bacterial colonization tests could be related to the much higher solution amount with which the coating has been contacted: 0.1 ml for the adhesion tests against 1 ml for the latter. This different amount could result in a higher dilution of the coating antibacterial mechanisms (for instance, the metal ion release or the direct bacteria/surface contact). From the point of view of the coating roughness, the measured RMS values are both falling within the range in which nanostructured antibacterial films of TiO_2_ obtained with the same synthesis technique present a linear increase in the bacteria adhesion (Singh et al., 2011), thus living the question open on the role of the RMS in such multielement films.

The non-specific mechanism of action, however, represents a tangible threat not only for microorganisms but also for eukaryotic cells, due to the ability of NPs to bind and permeate cells and consequently accumulate in biological fluids and tissue when used unconjugated (Sohaebuddin et al., 2010).

In light of this, in the present study, the cytotoxic potential was determined following the ISO 10993-10 Standard (ISO10993-10: 2010). According to these guidelines, if the cell viability is 70% lower than the negative control, the analyzed sample has a cytotoxic potential. Results obtained in the present experimental setting, demonstrated viability of 95.8% and 89.4% of cells cultured in the presence of Ti-Ag-Cu and Mg-Ag-Cu discs, respectively, when compared to negative controls. Comparing our data with those obtained and reported in the work of Benetti et al., 2019 on similar NP-coating, there was a notable improvement in biocompatibility properties in the new formulations analyzed. Indeed, the modulation of multi-element components by controlling the concentration, NPs size and shapes, or the surface area actively influences the biocompatibility characteristics and consequently might have an impact on the antibacterial properties.

Henceforward, investigations should take into account also the impact of a different percentage of elements of NPs in the modulation of biofilm formation over time. Furthermore, bacterial behavior is largely influenced by both surface alterations and the presence of substances in the physiological environment in which they grow, especially in their sessile form. Indeed, the proteome of the surrounding biological fluids might alter the physical and chemical characteristics of the surfaces of implanted materials (Großner-Schreiber et al., 2001). Thus, in all the reported analyses the bacteria culture was carried out in BHI broth supplemented with 50% v/v sterile human serum to try to mimic the influence of the physiological environment. Even though, the *in vitro* nature of all the analyses represents a known limitation of this study. Lastly, the promising anti-adhesive features of both Ti-Ag-Cu and Mg-Ag-Cu coatings, as well as their action in hampering the biofilm formation, should be tested on a wider panel of clinical isolates to further confirm the promising results obtained from these evaluations.

## 5 Conclusion

In conclusion, the present study highlighted the potential use of multi-element families of nanoparticles as new strategies against bacterial attachment to the surface of biomedical implants. Indeed, the bioactive features of Ti-Ag-Cu and Mg-Ag-Cu coatings were effective in decreasing the adhesion of *Staphylococcus aureus*, *Staphylococcus epidermidis* and *Escherichia coli* strains involved in PJIs compared to uncoated controls, regardless of their antibiotic resistance traits. The promising results were further supported by the investigation of biofilm formation upon coatings and the evaluation of bacterial colonization on nanoparticle-coated discs through confocal microscopy. A milder effect was detected against *P. aeruginosa* clinical isolates, probably due to the extraordinary capability of the strain in adhering to abiotic surfaces and deposing a thick slimy matrix on it.

Therefore, these investigations paved the way for future studies to achieve a correct balance between the anti-adhesive and anti-biofilm effects and cytotoxicity, by modulating the different percentage of elements of NPs in order to broaden the effect also against fastidious bacteria such as *P. aeruginosa*.

## Data availability statement

The datasets presented in this study can be found in online repositories. The names of the repository/repositories and accession number(s) can be found below: The datasets generated for this study can be found in the Zenodo repository at the following link: DOI: 10.5281/zenodo.7347618.

## Author contributions

MB, LG and EDV conceived and designed the study. MB, AL, VB, LC, and LR performed the experiments. MB, GT, LG, VB, and LR analyzed the data. MB and LG prepared figures and graphs and wrote the manuscript. LG and EDV critically revised the manuscript. All authors contributed to the article and approved the submitted version.
